# Socioeconomic, demographic and lifestyle-related factors associated with unhealthy diet: a cross-sectional study of university students

**DOI:** 10.1186/s12889-018-6149-3

**Published:** 2018-11-07

**Authors:** Roberto Martinez-Lacoba, Isabel Pardo-Garcia, Elisa Amo-Saus, Francisco Escribano-Sotos

**Affiliations:** 1 Facultad de Ciencias Económicas y Empresariales, Universidad de Castilla-La Mancha (UCLM), Plaza de la Universidad, 1 C.P.: 02071, Albacete, Spain; 2 Centro de Estudios Sociosanitarios (CESS), Universidad de Castilla-La Mancha (UCLM), Albacete, Spain

**Keywords:** Unhealthy diet, Socioeconomic factors, Demographic factors, lifestyle related factors, University students

## Abstract

**Background:**

Food habits are important to promote and maintain good health throughout life, and unhealthy diet is a modifiable and preventable risk factor. University students are a key group of adults among whom to promote healthy lifestyles. The aim of this study is to determine the factors associated with unhealthy diet in a sample of university students.

**Methods:**

An electronic cross-sectional survey was conducted with university students (*n*=593) from inland Spain, during the 2016-2017 academic year. The survey collected information on the participants’ food habits using an annual food frequency questionnaire. We also collected socioeconomic and demographic data and lifestyle-related information. A multivariate logistic regression was used for analysis.

**Results:**

The multivariate logistic regression revealed that the factors associated with an unhealthy diet were: being male, being underweight compared to the reference category (normal weight), having a mother of low socioeconomic status, the family home not being in the university city and, finally, studying a non health-related course

**Conclusion:**

Among students of the University of Castilla-La Mancha, being male, being underweight, the family home not being in the university city, having a mother of low socioeconomic status, and, finally, not studying a health-related course are the factors associated with a lower quality diet. Following a healthy diet is key in reducing the health costs of non-communicable diseases, and ensuring an acceptable long-term quality of life in populations.

**Electronic supplementary material:**

The online version of this article (10.1186/s12889-018-6149-3) contains supplementary material, which is available to authorized users.

## Background

Food habits are important to promote and maintain good health throughout life [1]. An unhealthy diet is a modifiable and preventable risk factor, which, together with other elements such as physical inactivity, use of tobacco and other harmful substances, has led to non-communicable chronic diseases (NCDs) becoming the leading cause of disability and early death, impacting on quality of life and the organization of healthcare systems [2, 3]. The global impact of NCD on healthcare expenditure is predicted to rise constantly over the coming years [4], thus representing a risk not only for human health but also for development and economic growth [5], since diet quality is influenced by socioeconomic position [6].

The Mediterranean diet is healthy and greater adherence to this diet has beneficial effects on health and can, among other things, ease the economic burden on the healthcare sector [7–11]. Although this diet is originally from the countries of the Mediterranean basin [12], thanks to its declaration as part of the Intangible Cultural Heritage of Humanity by UNESCO [13], and in view of the health benefits its provides, this food pattern has been exported to other countries [14, 15].

University students are a key group of adults among whom to promote healthy lifestyles [16], as they do not generally have good eating habits [17]. University courses can be used for improving food patterns [18], with the aim of reducing the social and health risks associated with an unhealthy diet. However, interventions and policies should study implementation conditions before application [19]. Previous studies in university population have used various indicators to descriptively analyse eating habits and diet quality [20–22], and the association between diet quality and lifestyle-related factors, social, economic and demographic characteristics [16, 23–25]. To the best of our knowledge, no study conducted with Spanish university students has studied unhealthy diets using an adherence to Mediterranean diet score as an indicator [26], and its association with lifestyle-related factors and socioeconomic and demographic characteristics. The aim of this study is to determine the factors associated with an unhealthy diet, so as to assist decision makers in promoting food and eating policies which reduce the risk of NCDs and the associated economic problems, thus ensuring enhanced quality of life among the population.

## Method

### Design

This study was conducted in the Autonomous Community of Castilla-La Mancha, situated in the centre of Spain. Students from the University of Castilla-La Mancha (a multi-campus institution) in the cities of Albacete, Ciudad Real, Cuenca, Talavera de la Reina and Toledo participated in the study. An electronic self-completed cross-sectional survey conducted during the 2016-2017 academic year was used to gather data. The students could ask researchers for assistance if they had any doubts. All the students were informed of the aims of the study and participated voluntarily. The survey included questions about socioeconomic, demographic, anthropometric characteristics, physical activity, and food intake. A pre-test was performed during the 2015-2016 academic year to confirm face and content validity of the survey. Items were refined according to the comments from the pre-test. The data were collected using the Survey Monkey software [27].

### Participants and environment

**Fig. 1:**
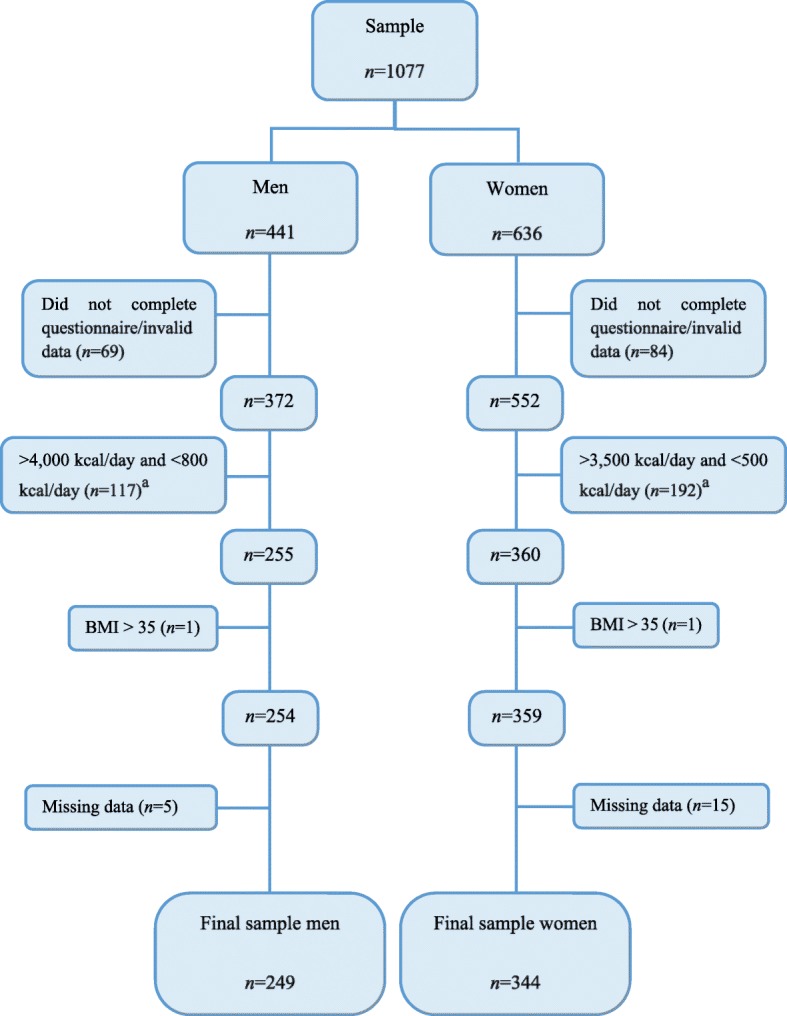
Data cleaning process

A total of 1077 students participated in the study (*n*=1077). The final sample comprised 593 participants (*n*=593, 249 men and 344 women). This sample was representative of the study population (15,278 students, 3971 Health Sciences and 11,307 Social Sciences), and the CI was established at 95% with an estimated maximum sampling error of ± 4%. The inclusion criterion was that participants were enrolled on Social Sciences courses (i.e.: Business & Administration, Economics, Law & Economics…) or Health Sciences courses (i.e.: Nursing, Medicine, Pharmacy…) during the 2016-2017 academic year in the University of Castilla-La Mancha. On the other hand, participants were excluded if: i) did not complete the questionnaire/invalid data (i.e.: careless or insufficient effort); ii) energy intake under/over limit; iii) BMI > 35; iv) missing data. Figure 1 shows the data cleaning process.

### Dietary assessment

We used a self-reported annual Food Frequency Questionnaire (FFQ) to collect data on food intake. The FFQ was adapted from similar FFQs previously validated in Spanish adult population [28–31]. Participants were asked about their consumption of 141 foods divided into 12 groups: i) dairy products; ii) eggs, meat and fish; iii) vegetables; iv) legumes; v) cereal; vi) oils and fats; vii) fruit; viii) sweets and desserts; ix) beverages; x) spices; xi) precooked products; and xii) fast food. Energy intake was calculated by multiplying the frequency of consumption in years, the grams per portion and the kilocalories (kcal) for each food (Additional file 1: Table S1). The energy intake for each type of food was taken from the Spanish Food Composition Database [32].

### Variables included

#### Socioeconomic and demographic characteristics

The socioeconomic and demographic characteristics collected were gender, age, family home, parents’ current employment, degree in which the students were enrolled, type of residence during the academic year (family home without cooking; university residence without cooking; shared flat with cooking; and shared flat without cooking).

The parents’ occupational social class (mother and father) was established following the 4^th^ Spanish National Classification of Occupations using two categories [33]. Occupational social class was considered an indicator of socioeconomic status [34, 35]. Participants whose parents belonged to the group of non-manual workers were associated with high socioeconomic status and those whose parents belonged to the group of manual workers were considered of low socioeconomic status (Additional file 1: Table S2).

We differentiated between students enrolled on health-related degrees (Medicine, Nursing or Pharmacy) and Social Sciences related degrees. Respondents who reported living in a shared flat and cooking were considered to be independent and to cook regularly for themselves.

#### Lifestyle-related factors

Self-reported height and weight were collected. Body mass index (BMI) was calculated using anthropometric data (kg/m^2^) and classified into 4 categories: underweight (BMI < 18.5); normal weight (18.5 ≤ BMI ≤ 24.9); overweight (25 ≤ BMI ≤ 29.9) and obesity (BMI ≥ 30) [36]. The participants were considered to present a healthy level of physical activity if they accumulated at least 60 minutes of moderate-intensity physical activity daily (brisk walking, trekking, bicycling, swimming, basketball or volleyball) and 30 minutes of intense physical activity daily (running, mountain biking, singles tennis, football, or aerobic exercise) or a combination of both [37, 38]. Tobacco use, and consumption of other harmful substances (i.e.: cannabis, cocaine, amphetamines…) were also included in the questionnaire.

#### Unhealthy diet

We defined unhealthy diet by using an index of adherence to Mediterranean diet. We used the FFQ to estimate adherence to Mediterranean diet according to the validated MEDI-LITE score [7, 26]. This indicator comprises nine food categories: fruit, vegetables, legumes, cereal grains, fish, meat and meat products, dairy products, alcohol, and olive oil. The possible score ranges from 0 (minimum) to 18 (maximum). We converted the index into a dichotomous variable named unhealthy diet, using the median as the reference [39]. Diet was considered unhealthy (score 1) if the score on the index was ≤ 9 (median), and adequate if it was > 9 (score 0) (Additional file 1: Table S3).

### Missing data analysis

Multiple imputation procedure was performed to deal with missing data, under the missing at random assumption (MAR) [40, 41]. We excluded from analyses all participants who did not complete the full questionnaire or presented invalid data (*n*=153). Participants were included in multiple imputation analyses if they completed the questionnaire, despite they presented extreme values (i.e.: overreport of kilocalories/day, BMI>35) or missing values. Extreme values were considered as missing data. Variables with missing data were: unhealthy diet (32.14%), his/her father’s socioeconomic status (2.60%), his/her mother’s socioeconomic status (0.008%) and body mass index (0.002%). We compared observed and imputed data for unhealthy diet variable, because there is a large fraction of imputed data [42] (Additional file 1: Table S6). Variables included in imputation model were presented in Additional file 1. We performed two multiple imputation analyses with 5 and 30 subsets (number of iterations=20). We also analysed interactions in imputation models (Additional file 1: Table S5).

### Statistical analysis

The statistical model used was multivariate logistic regression. The dichotomous dependent variable was unhealthy diet, created using the MEDI-LITE index. The independent variables were related to: a) lifestyle: body mass index, level of physical activity, tobacco use, and use of harmful substances; and b) socioeconomic and demographic characteristics: socioeconomic status of father and mother, main family home, whether or not the participant cooked for him or herself during the academic year, and the subject area of the degree course. We analysed the interactions between the variables included in the study (Additional file 1: Table S4). The correlations were analysed using Spearman’s Correlation Coefficient [43]. All the statistical analyses were conducted using RStudio software [44], and Microsoft Excel spreadsheet software [45].

## Results

**Fig. 2:**
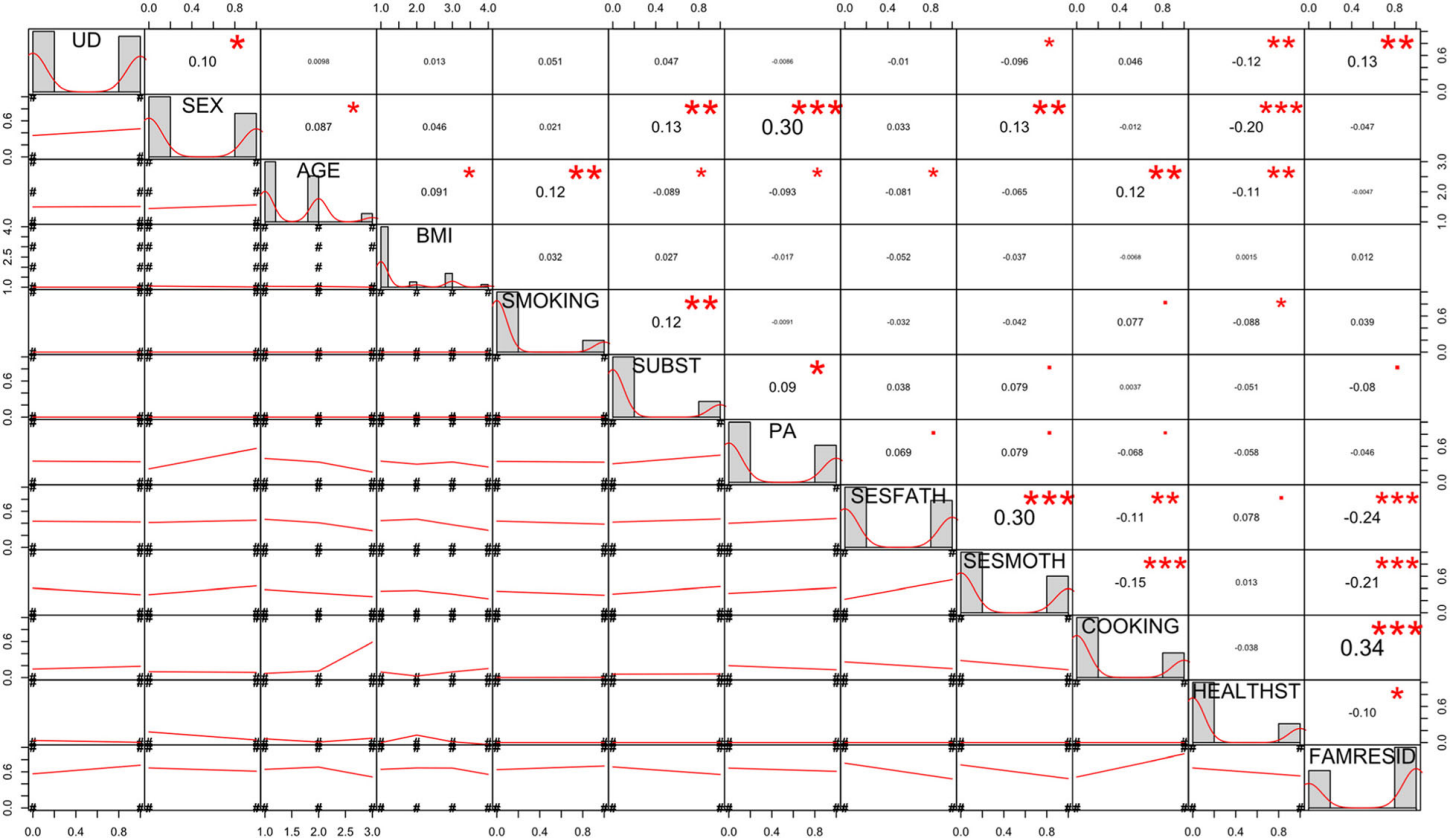
Correlation matrix between variables. Abbreviations: UD unhealthy diet, BMI body mass index, SUBST substance use, PA physical activity, SESFATH father’s socioeconomic status, SESMOTH mother’s socioeconomic status, HEALTHST health-related studies, FAMRESID family home

The main diagonal of the correlation matrix (Fig. 2) shows graphically the distribution of the variables. Table 1 shows the characteristics of the sample (*n*=593) according to gender.

**Table 1:** Characteristics of the study population

	Total (*n*=593)	Women (*n*=344)	Men (*n*=249)	
	%^a^	%^a^	%^a^	*P value*
Unhealthy diet	47.90	150 (43.60)	134 (53.81)	0.018
*a) Socioeconomic and demographic characteristics*				
Age (years), mean	20.21	20.06	20.42	0.176
SD	3.23	3.25	3.21	
SES father/ mother				
High	43.84 / 37.60	42.44 / 32.27	45.78 / 44.98	0.560/0.004
Low	56.16 / 62.40	57.56 / 67.73	54.22 / 55.02	
Family home				
Other town	61.72	63.66	59.04	0.290
University city	38.28	36.34	40.96	
Cooks for him or herself during the academic year				
Yes	29.17	29.65	28.51	0.834
No	70.83	70.35	71.49	
Degree course				
Health Sciences	23.95	31.10	14.06	<0.001
Social Sciences	76.05	68.90	85.94	
*b) Lifestyle-related factors*				
Weight (kg) mean	66.02	59.54	74.96	<0.001
SD	12.65	10.04	10.18	
Height (cm) mean	169.80	164.01	177.80	<0.001
SD	9.23	5.99	6.55	
BMI				
Underweight	6.41	10.17	1.20	<0.001
Normal weight	73.52	74.42	72.29	0.627
Overweight	16.86	11.92	23.70	<0.001
Obesity	3.21	3.49	2.81	0.821
HPA				
Yes	38.11	25.58	55.42	<0.001
No	61.89	74.42	44.58	
Tobacco use				
Yes	16.36	15.70	17.27	0.691
No	83.64	84.30	82.73	
Other harmful substances				
Yes/occasional	20.57	15.99	26.91	0.002
No	79.43	84.01	73.09	

Gender related-differences between means or percentages calculated using the *t*-Student test and χ^2^

*HPA*, healthy physical activity, *BMI* body mass index, *SES* socioeconomic status.

^a^Values presented are percentages, unless indicated otherwise.

The students’ mean age was 20.21 years and 58% of the respondents were female. The mean height was 169.80 and mean weight was 66.02 kg, with significant differences between men and women. A total of 47.90% of the students presented an unhealthy diet, with a greater difference in the men (53.81%). Most of the participants were normal weight (74.42% of women 72.29% of men). The underweight percentage was higher in the women (10.17%) and the overweight percentage was higher in the men (23.70%). The percentage of males who did healthy physical activity was higher (55.42% versus 25.58%).

Of the fathers, 43.84% had non-manual occupations (high socioeconomic status) compared to 37.60% of the mothers. These differences were greater when divided by gender, since 32.27% of female students’ mothers had a non-manual occupation, compared to 44.98% of male students’ mothers. A majority of students reported their family home was in different town from the university (61.72%), did not cook during the academic year (70.83%), and did not smoke (83.64%) or use harmful substances (79.43%), although male students reported higher consumption (occasional or regular) of substances (26.91% compared to 15.99% of females). A total of 23.95% of the respondents studied a health-related degree course although the difference was greater among the women (31.10% compared with 14.06% of the men).

The upper part of Fig. 2 shows that the highest correlations are only moderate (0.30<|ρ|<0.70). The lower part shows that the positive or negative relationships between the variables. Unhealthy diet is positively related to gender and family home, and negatively to the maternal socioeconomic status and the subject area of the student’s degree course.

The results of the multivariate logistic regression analysis for the associations between the sociodemographic and lifestyle-related factors and unhealthy diet as dependent variable are shown in Table 2. Table 2 includes the results from complete-case analysis and multiple imputation analyses (m=5 and m=30). The results from both analyses produce similar results for odds ratio on studied variables and complete-case analysis showed comparable, but higher standard error than multiple imputation analysis (Additional file 1: Table S7). The factors associated with unhealthy diet were: being male, being underweight relative to the category of reference category (normal weight), low maternal socioeconomic status, the participant’s family habitually residing in a town away from the university and, finally, studying a health-related degree course.

**Table 2:** Association between socioeconomic, demographic and lifestyle-related factors and unhealthy diet: multivariate logistic regression analysis

Variables	Complete-case analysis (*n*=593)		Multiple imputation (*n*=924, m=5)		Multiple imputation (*n*=924, m=30)	
	OR	95% CI	OR	95% CI	OR	95% CI
*a) Socioeconomic and demographic*						
Age						
15-19	1.00	Reference				
20-24	1.00	(0.70; 1.43)	0,93	(0.67; 1.28)	1.02	(0.73; 1.42)
25-25+	0.92	(0.46; 1.84)	0,97	(0.51; 1.84)	0.95	(0.53; 1.70)
Gender						
Woman	1.00	Reference				
Man	1.75*	(1.20; 2.55)	1.80*	(1.26; 2.57)	1.78*	(1.26; 2.51)
SES father						
Low	1.00	Reference				
High	1.24	(0.86; 1.78)	1.22	(0.88; 1.68)	1.21	(0.86; 1.70)
SES mother						
Low	1.00	Reference				
High	0.63*	(0.44; 0.92)	0.63*	(0.45; 0.89)	0.64*	(0.45; 0.92)
Family home						
University city	1.00	Reference				
Other town	1.69*	(1.15; 2.48)	1.89*	(1.36; 2.61)	1.69*	(1.19; 2.41)
Cooks for him or herself during the academic year						
No	1.00	Reference				
Yes	0.98	(0.66; 1.45)	0.90	(0.61; 1.31)	0.99	(0.68; 1.46)
Degree course						
Social Sciences	1.00	Reference				
Health Sciences	0.63*	(0.42; 0.95)	0.59*	(0.41; 0.87)	0.65*	(0.46; 0.93)
*b) Lifestyle-related*						
BMI						
Normal weight	1.00	Reference				
Underweight	2.31*	(1.14; 4.82)	2.19*	(1.07; 4.45)	2.28*	(1.16; 4.46)
Overweight	0.81	(0.51; 1.28)	0.74	(0.43; 1.26)	0.78	(0.50; 1.22)
Obesity	1.04	(0.39; 2.74)	1.04	(0.43; 2.52)	1.03	(0.42; 2.51)
HPA						
No	1.00	Reference				
Yes	0.82	(0.57; 1.18)	0.82	(0.60; 1.11)	0.80	(0.57; 1.13)
Tobacco use						
No	1.00	Reference				
Yes	1.20	(0.76; 1.90)	1.20	(0.78; 1.83)	1.21	(0.79; 1.86)
Other harmful substances						
No	1.00	Reference				
Yes/occasional	1.29	(0.84; 1.97)	1.33	(0.91; 1.95)	1.28	(0.86; 1.91)

*Abbreviations*: *BMI* body mass index, *HPA* healthy physical activity, *SES* socioeconomic status

*p<0.05. Complete-case analysis, multiple imputation where m= number of subsets.

## Discussion

The results of this work show that the factors associated with low-quality diet are: gender, location of family home, body mass index, mother’s socioeconomic status and subject area of studies. Age, tobacco or substance use, and cooking during the academic year are not associated with unhealthy diet. These findings are important for public health. Our results help to identify the characteristics of individuals requiring intervention to encourage a healthy diet. Non-communicable diseases such as cardiovascular disease, type 2 diabetes mellitus, and cancer account for about 10% of direct medical costs [46], and are lifestyle-related [47]. Diet and nutrition, among others, are important factors in health promotion, and primary prevention is an effective and affordable way to prevent chronic disease [48]. Hence, early recognition of unhealthy diets in youths could help to avoid the healthcare utilisation and costs associated with non-communicable diseases [4], and could also improve quality of life.

The characteristics of the variables included in this study are similar to those used in various studies in university population [20, 49–52]. In line with other studies [53, 54], our work also reports a greater prevalence of underweight in women and overweight in men. As regards the socioeconomic and demographic characteristics, the results reveal that age is not a significant factor in diet quality [51, 55], although other studies have found an association [23, 24]. It is worth remembering, however, that studies in university populations refer to a small age range and, so, the age variable may have little impact on diet quality.

The female participants presented higher diet quality, coinciding with various studies in university population [24, 51, 52], and in adult population [56, 57]. Nonetheless, there exists one work in an adult population showing higher diet quality in men [58], contrasting with our results. In the present work, higher maternal socioeconomic status was inversely related to unhealthy diet. Coinciding with our study, a number of studies in adult population have shown that diet quality is associated with higher socioeconomic status [59–63], and in an adolescent population (13-19 years), higher maternal socioeconomic status, measured by means of educational level, was positively associated with better dietary habits [64].

Our findings also show that when the family home was not in the university city, the participant did not live at home and, consequently, presented a less healthy diet. In contrast, when a student’s family lives in the university city, the participant usually lives in the family home and thus maintains better dietary patterns [52]. Whether participants cooked for themselves or not was not associated with diet quality, in contrast to another study showing a relationship between cooking meals for oneself and higher diet quality [65]. Finally, studying a health-related degree course was associated with higher diet quality [50, 66]. In contrast, there is one work showing that knowledge of nutrition does not affect diet and healthy lifestyle decision-making [67].

Regarding lifestyle-related factors, our results show that underweight is associated with lower diet quality compared to normal weight participants. Being overweight and obesity were not associated with diet quality, coinciding with various studies in university population [20, 23, 24, 55]. This, however, contrast with findings in adult population [58, 60]. On the other hand, there exists one study in student population finding that overweight and obesity are associated with lower diet quality [51]. These differences in findings about the association between BMI and diet quality may be a result of participants’ social desirability bias.

Several studies have shown that smoking [56, 60, 62], and low levels of physical activity are factors associated with an unhealthier dietary pattern [56, 57, 60, 62, 68]. However, in student population there is no consensus on whether smoking and doing healthy physical activity are associated with diet quality. In our smoking and physical activity were not associated with unhealthy diet [20, 51, 52, 55], although one study has shown that physical activity is associated with healthier dietary patterns [24]. Finally, the present work finds no association between the consumption of harmful substances and diet quality [24].

This study has some limitations. First, the use of a FFQ may give rise to dietary measurement error [69], impacting on the estimation of relative risks. Furthermore, given the characteristics of food frequency questionnaires, a memory bias might have influenced the results. A social desirability bias might also have occurred, whereby respondents reported healthy habits which did not exist. The collection of weight and height was self-reported. However, a recent study has shown that self-reported anthropometric data are valid in young adults [70]. Nevertheless, the study also has some strengths. We also used the criterion of recommended intake in kilocalories [71], which presents no substantial differences to other methods [72], to exclude participants with implausible energy intakes. We dealt with missing data by using a multiple imputation method. Imputed values on unhealthy diet variable were comparable with the observed values. Focusing on multivariate logistic regression, this method produced similar estimates on odd ratio for unhealthy diet than a complete-case analysis and a comparable standard error.

## Conclusion

This study identifies factors associated with an unhealthy diet. The results showed that being male, underweight, the family home not being in the university city, low maternal socioeconomic status and not studying a health-related degree course are all factors associated with low diet quality. Maintaining a healthy diet is of great importance in reducing health costs related to non-communicable diseases and ensuring acceptable long-term quality of life. The findings of the present study may serve to develop food policies that help to promote healthy lifestyles in the student population.

## Additional file

Additional file 1: Data cleaning, calculation of variables, statistical analysis; **Table S1.** Conversion of intake frequencies to number of intakes per year; **Table S2.** Occupational social class based on the occupations in the questionnaire; **Table S3.** Adjustment of daily/weekly frequencies; **Table S4.** Association between socioeconomic, demographic and life-style related factors and unhealthy diet: multivariate logistic regression with interaction of variables; **Table S5.** Interaction effects in imputation models (n=924): multivariate logistic regression; **Table S6.** Comparison between observed data and imputed data for unhealthy diet variable; **Table S7.** Coefficients and standard error from multivariate logistic regression. (DOCX 61 kb)

## Abbreviations

BMI: Body mass index
FAMRESID: Family home
FFQ: Food frequency questionnaire
HEALTHST: Health-related studies
NCD: Non-communicable chronic diseases
OR: Odd ratio
PA: Physical activity
SD: Standard deviation
SESFATH: Father's socioeconomic status
SESMOTH: Mother's socioeconomic status
SUBST: Substance use
UD: Unhealthy diet
