# Supplementary material for: Socioeconomic, demographic and lifestyle-related factors associated with unhealthy diet: a cross-sectional study of university students
Source: BMC Public Health. 2018 Nov 7;18:1241. doi: 10.1186/s12889-018-6149-3 (PMC6223081; doi:10.1186/s12889-018-6149-3)
Supplement: Supplementary file 1 — Data cleaning, calculation of variables, statistical analysis; Table S1. Conversion of intake frequencies to number of intakes per year; Table S2. Occupational social class based on the occupations in the questionnaire; Table S3. Adjustment of daily/weekly frequencies; Table S4. Association between socioeconomic, demographic and life-style related factors and unhealthy diet: multivariate logistic regression with interaction of variables; Table S5. Interaction effects in imputation models (n=924): multivariate logistic regression; Table S6. Comparison between observed data and imputed data for unhealthy diet variable; Table S7. Coefficients and standard error from multivariate logistic regression. (DOCX 61 kb) [file 12889_2018_6149_MOESM1_ESM.docx]

# Additional file 1

## Data cleaning

The data cleaning process reduced the sample from *n=*1077 participants to *n=*593. First, *n*=153 participants were excluded who did not complete the food frequency questionnaire. Second, *n=*309 participants were excluded who exceeded the maximum intake of kcal/day. Finally, *n=*22 participants who had missing data or extreme scores on BMI were excluded.

### Calculation of kilocalories and exclusion of participants

First, the kilocalories (kcal) provided by each type of food was obtained using the Spanish Food Composition Database (BEDCA) [1]. Next, all the intake frequencies were converted to numerical values interpreted as “number of intakes per year”, as shown in Table S1.

The calculation for daily frequencies was:

$$Number of intakes= \bar{X}_{daily} \times365$$

The calculation for weekly frequencies was:

$$Number of intakes=\bar{X}_{weekly} \times52$$

The calculation for monthly frequencies was:

$$Number of intakes=\bar{X}_{monthly} \times12$$

## Calculation of variables

### Social, economic and demographic characteristics: socioeconomic status

The questionnaire included the current parental occupation following the Spanish National Classification of Occupations (CNO-11), together with the following categories: non-working, retired, unemployed and civil servant. There was also an option to indicate a parent’s profession not included in the questionnaire. To analyse the social class according to occupation, we used the Grouped Classification IV (2 categories) [2]. Table S2 shows the occupations included in the questionnaire and the classification by occupation. If a participant reported their parent was self-employed, this parent was included in group 1.

### Lifestyle-related factors: level of healthy physical activity

The participants were considered to present a healthy level of physical activity if they accumulated at least 60 minutes of moderate-intensity physical activity daily (brisk walking, trekking, bicycling, swimming, basketball or volleyball) and 30 minutes of intense physical activity daily (running, mountain biking, singles tennis, football, or aerobic exercise) or a combination of both [3, 4]. Thus, we obtained the time for each activity, which were summed and grouped into moderate-intensity and intense physical activities. Of the *n=*593 participants included in the study, *n*=226 presented healthy physical activity.

### Food patterns and diet quality based on adherence to Mediterranean diet

To construct the unhealthy diet variable, we used the level of adherence to the Mediterranean diet [5], adjusting the results obtained from the food frequency questionnaire to the proposed, validated scale. The index (MEDI-LITE) is calculated according to number of daily/weekly portions, which are scored 0, 1 or 2 points for the nine groups of foodstuffs (fruit, vegetables, legumes, cereals, fish, meat and meat products, dairy products, alcohol and olive oil). Table S3 shows the conversion of the intake frequencies. We multiplied each intake by the weight in grams of each foodstuff. The different foods were summed, and the results were then adjusted for the number of each portion indicated by the index. If, for example, a hypothetical participant consumed 1 glass of semi-skimmed milk (200gr/glass) and 2.5 yogurts in one day (125gr/yogurt), the calculation was: (1 x 200) + (2.5 x 125) = 512.5 grams of dairy products. According to the index, a portion of dairy products is 180 grams, so 512.5/180 = 2.84 portions/day. Thus, the participant’s dairy product score would be 0: 2.84>1.5. Once the index score was obtained, it was dichotomized: diet was considered unhealthy (score 1) if the index value wads within the interval of 0-9 and was considered adequate within the interval of 10-18 (score 0). One limitation of the index is that it shows the recommended daily/weekly level of intake of the different food groups but does not express the maximum of these foods. We have attempted to overcome this limitation by excluding participants who exceeded the maximum number of daily kilocalories [6].

## Statistical analysis

### Linearity study of the quantitative variables

To incorporate a quantitative variable into a logistic regression model as an independent variable, its linearity with respect to the dependent variable must be determined [7]. To this end, we categorized the quantitative variable of age (*AGE).*  Age was defined in three intervals: 15-19, 20-24, 25-25+. The *AGE* variable should not be introduced as a quantitative variable as no linear relationship is revealed. An explanation for this absence of linearity may be that the university students in the first group are younger and thus more dependent on the food from the family home or the food served at their halls of residence.

### Categorization of the variables

The variables were categorized as follows: unhealthy diet (unhealthy = 1; adequate = 0), age (15-19 = 1; 20-24 = 2; 25-25+ = 3), gender (male = 1; female = 0), BMI (normal weight = 1; underweight = 2; overweight = 3; obesity = 4), healthy physical activity (yes = 1; no = 0socioeconomic status father/mother (high = 1; low = 0), family home (other town = 1; university city = 0), cooks for him or herself during the academic year (yes = 1; no = 0), tobacco use (yes = 1; no = 0), substance use yes/occasional= 1; no = 0), degree course (health-related = 1; non-health-related = 0).

### Multivariate logistic regression model

The multivariate logistic regression model [7–9], is, generally speaking, presented as follows:

$$\ln\left( \frac{p}{1-p} \right)=a+b_{1}x_{1}+b_{2}x_{2}+\ldots+b_{p}x_{p}$$

In our case, the dependent variable is unhealthy diet (UD). It is dichotomous and constructed using the Mediterranean diet adherence index (MEDI-LITE) [10]. The variables included are: a) socioeconomic and demographic variables: socioeconomic status associated with the occupational social class of both the father (*SESFATH*) and the mother (*SESMOTH*), whether the family home is in the university city or another town (*FAMHOME*), whether the participant cooks for him or herself during the academic year (*COOKING*), whether the participant studies a health-related degree course or not *(STUDIES*); b) lifestyle-related factors: body mass index (*BMI*), tobacco use (*SMOKING*), use of other harmful substances (*SUBST*), healthy physical activity (*PA*). We also studied possible interactions between the variables and if these were significant, they were included in the final model. The model was adjusted for age and gender. The interactions analysed were: *COOKING*FAMRESID* y *SMOKING*SUBST.* None of the interactions was significant and were thus not included in the proposed model. The results for the model without interactions are presented in the original model and the results derived from the model with interactions (coefficient and p-value) are shown in Table S4. The interaction between the variables does not significantly alter the results obtained, ruling out the possibility of interaction. All the statistical analyses were conducted using RStudio software [11], and Microsoft Excel spreadsheet program [12].

### Missing data and multiple imputation

We performed a multiple imputation model to deal with missing data and we made the missing at random assumption [13–15]. We included these variables in the imputation model: body mass index, level of physical activity, tobacco use, and use of harmful substances, socioeconomic status of father and mother, main family home, whether or not the participant cooked for him or herself during the academic year, and the subject area of the degree course. We also studied interaction effects in the imputation models (Table S5). We compared observed and imputed data for unhealthy diet variable (Table S6). There are no big differences between observed and imputed data for unhealthy diet variable. Table S7 shows coefficients and standard error for a complete-case analysis and a multiple imputation analyses (m=5 and m=30 subsets).

### Goodness of fit

Different statistics may be used to verify the goodness of fit [16]. This work uses the following tests: Hosmer-Lemeshow (*C_g_*=0.55), Hosmer-Lemeshow (*H_g_*=0.35) Cessie and Van Houwelingen ($\hat{T}$*_lc_*=0.84) and Stukel (*Ŝ_ST_*=0.20). In the four cases, the null hypothesis of the good fit is verified with a significance level of 0.05.

# References

1. Red BEDCA, Agencia Española de Seguridad Alimentaria y Nutrición. Base de Datos Española de Composición de Alimentos. 2010. http://www.bedca.net/. Accessed 7 Oct 2017.

2. Domingo-Salvany A, Bacigalupe A, Carrasco JM, Espelt A, Ferrando J, Borrell C. Propuestas de clase social neoweberiana y neomarxista a partir de la Clasificación Nacional de Ocupaciones 2011. Gac Sanit. 2013;27:263–72. doi:10.1016/j.gaceta.2012.12.009.

3. Grupo Colaborativo de la Sociedad Española de Nutrición Comunitaria. Guías alimentarias para la población española; la nueva pirámide de la alimentación saludable. Nutr Hosp. 2016;33 Suplemento 8:1–48. doi:10.20960/nh.827.

4. World Health Organization. Global Recommendations on Physical Activity for Health. Geneva: World Health Organization; 2010. http://www.who.int/dietphysicalactivity/factsheet_recommendations/en/.

5. Sofi F, Dinu M, Pagliai G, Marcucci R, Sofi F, Dinu M, et al. Validation of a literature-based adherence score to Mediterranean diet: the MEDI-LITE score. Int J Food Sci Nutr. 2017;68:757–62. doi:10.1080/09637486.2017.1287884.

6. Willett W. Nutritional epidemiology. Oxford: Oxford University Press; 1998.

7. Martínez-González MA, Sánchez-Villegas A, Toledo Atucha EA, Faulin Fajardo J. Bioestadística amigable. Barcelona: Elsevier España; 2014.

8. Mangiafico SS. An R Companion for the Handbook of Biological Statistics, version 1.3.2. 2015. rcompanion.org/documents/RCompanionBioStatistics.pdf.

9. McDonald JH. Handbook of Biological Statistics. 3rd edition. Baltimore, Maryland, U.S.A.: Sparky House; 2009.

10. Sofi F, Macchi C, Abbate R, Gensini GF, Casini A. Mediterranean diet and health status: an updated meta-analysis and a proposal for a literature-based adherence score. Public Health Nutr. 2013;17:2769–82. doi:10.1017/S1368980013003169.

11. RStudio Team. RStudio: Integrated Development for R. 2015. http://www.rstudio.com/.

12. Microsoft. Excel 2016. 2016. https://products.office.com/es-es/excel.

13. Buuren S van, Groothuis-Oudshoorn K. mice: Multivariate Imputation by Chained Equations in R. J Stat Softw. 2011;45. doi:10.18637/jss.v045.i03.

14. Sterne JAC, White IR, Carlin JB, Spratt M, Royston P, Kenward MG, et al. Multiple imputation for missing data in epidemiological and clinical research: potential and pitfalls. BMJ. 2009;338 b:2393:157–60.

15. Pedersen AB, Mikkelsen EM, Cronin-Fenton D, Kristensen NR, Pham TM, Pedersen L, et al. Missing data and multiple imputation in clinical epidemiological research. Clin Epidemiol. 2017;9:157–66.

16. Hosmer DW, Hosmer T, Le Cessie S, Lemeshow S. A comparison of goodness-of-fit tests for the logistic regression model. Stat Med. 1997;16:965–80.

Table S1. Conversion of intake frequencies to number of intakes per year

| **Intake frequencies** | **Number of intakes per year** |
| --- | --- |
| Never or hardly ever | 0 |
| One unit per day | 365 |
| 2 to 3 units per day | 913 |
| 4 to 5 units per day | 1643 |
| 6 or more units per day | 2190 |
| 1 to 2 units per week | 78 |
| 3 to 4 units per day | 182 |
| 5 or more units per week | 260 |
| 1 to 3 units per month | 24 |

Table S2. Occupational social class based on the occupations in the questionnaire

|  | Occupation | Occupational social class^a^ | |
| --- | --- | --- | --- |
| 1 | Management in companies and the public administration | I | |
| 2 | Scientific and cultural technicians and professionals |  |  |
| 3 | Clerical workers | I | |
| 4 | Support technicians and professionals |  |  |
| 5 | Hospitality and retail workers | II | |
| 6 | Armed forces | I | |
| 7 | Mining, construction and manufacturing workers | II | |
| 8 | Agricultural and fishing workers |  |  |
| 9 | Unskilled workers |  |  |
| 10 | Non-working |  |  |
| 11 | Retired |  |  |
| 12 | Unemployed |  |  |
| 13 | Civil servant | I | |
| ^a^ Domingo-Salvany et al. (2013)[2]  I, non-manual workers; II, manual workers | | |  |

Table S3. Adjustment of daily/weekly frequencies

| Intake frequencies | Number of intakes per day | Number of intakes per week |
| --- | --- | --- |
| Never or hardly ever | 0 | 0 |
| One unit per day | 1 | 7 |
| 2 to 3 units per day | 2.5 | 14 |
| 4 to 5 units per day | 4.5 | 14 |
| 6 or more units per day | 6 | 14 |
| 1 to 2 units per week | 0.21 | 1.5 |
| 3 to 4 units per day | 0.5 | 3.5 |
| 5 or more units per week | 0.71 | 5 |
| 1 to 3 units per month | 0 | 0 |

Table S4. Association between socioeconomic, demographic and life-style related factors and unhealthy diet (n=593): multivariate logistic regression with interaction of variables

| Variables |  | Coefficient | SE | Wald | P value |
| --- | --- | --- | --- | --- | --- |
| *a) Socioeconomic and demographic* |  |  |  |  |  |
| Age | 15-19 | Reference |  |  |  |
|  | 20-24 | -0.00 | 0.18 | -0.02 | 0.988 |
|  | 25-25+ | -0.03 | 0.37 | -0.08 | 0.932 |
| Gender | Woman | Reference |  |  |  |
|  | Man | 0.56 | 0.19 | 2.84 | 0.004 |
| SES father | Low | Reference |  |  |  |
|  | High | 0.19 | 0.18 | 1.01 | 0.312 |
| SES mother | Low | Reference |  |  |  |
|  | High | -0.44 | 0.19 | -2.32 | 0.013 |
| Family home | University city | Reference |  |  |  |
|  | Other town | 0.43 | 0.21 | 2.03 | 0.042 |
| Cooks for him or herself during the academic year | No | Reference |  |  |  |
|  | Yes | -0.48 | 0.52 | -0.94 | 0.350 |
| Degree course | Non health-related | Reference |  |  |  |
|  | Health related | -0.47 | 0.21 | -2.18 | 0.027 |
| *b) Lifestyle-related* |  |  |  |  |  |
| BMI | Normal weight | Reference |  |  |  |
|  | Underweight | 0.83 | 0.36 | 2.29 | 0.022 |
|  | Overweight | -0.20 | 0.23 | -0.87 | 0.383 |
|  | Obesity | 0.03 | 0.49 | 0.06 | 0.951 |
| HPA | No | Reference |  |  |  |
|  | Yes | -0.22 | 0.19 | -1.20 | 0.229 |
| Tobacco use | No | Reference |  |  |  |
|  | Yes | 0.15 | 0.28 | 0.54 | 0.593 |
| Substance use | No | Reference |  |  |  |
|  | Yes/occasional | 0.20 | 0.25 | 0.79 | 0.429 |
| *c) Interactions* |  |  |  |  |  |
| Cooks during the academic year x Family home | Doesn’t cook x city | Reference |  |  |  |
|  | Cooks x village | 0.56 | 0.56 | 1.00 | 0.316 |
| Tobacco use x Substance use | Doesn’t smoke x doesn’t use substances | Reference |  |  |  |
|  | Smokes x uses substances | 0.22 | 0.52 | 0.41 | 0.680 |
| BMI, body mass index; HPA, healthy physical activity; SE, standard error; SES, socioeconomic status. | | | | | |

Table S5. Interaction effects in imputation models (n=924): multivariate logistic regression with interaction of variables

|  |  | Multiple imputation (m=5) | | Multiple imputation (m=30) | |
| --- | --- | --- | --- | --- | --- |
| Variables |  | Coefficient | P value | Coefficient | P value |
| *a) Socioeconomic and demographic* |  |  |  |  |  |
| Age | 15-19 | Reference |  |  |  |
|  | 20-24 | -0.08 | 0.628 | 0.02 | 0.910 |
|  | 25-25+ | 0.01 | 0.993 | -0.02 | 0.943 |
| Gender | Woman | Reference |  |  |  |
|  | Man | 0.57 | 0.002 | 0.57 | 0.001 |
| SES father | Low | Reference |  |  |  |
|  | High | 0.20 | 0.231 | 0.19 | 0.282 |
| SES mother | Low | Reference |  |  |  |
|  | High | -0.45 | 0.010 | -0.44 | 0.015 |
| Family home | University city | Reference |  |  |  |
|  | Other town | 0.56 | 0.002 | 0.47 | 0.021 |
| Cooks for him or herself during the academic year | No | Reference |  |  |  |
|  | Yes | -0.50 | 0.254 | -0.31 | 0.479 |
| Degree course | Non health-related | Reference |  |  |  |
|  | Health related | -0.53 | 0.007 | -0.44 | 0.017 |
| *b) Lifestyle-related* |  |  |  |  |  |
| BMI | Normal weight | Reference |  |  |  |
|  | Underweight | 0.77 | 0.035 | 0.82 | 0.018 |
|  | Overweight | -0.30 | 0.273 | -0.25 | 0.279 |
|  | Obesity | 0.05 | 0.920 | 0.02 | 0.961 |
| HPA | No | Reference |  |  |  |
|  | Yes | -0.20 | 0.213 | -0.22 | 0.220 |
| Tobacco use | No | Reference |  |  |  |
|  | Yes | 0.09 | 0.732 | 0.17 | 0.486 |
| Substance use | No | Reference |  |  |  |
|  | Yes/occasional | 0.21 | 0.342 | 0.23 | 0.326 |
| *c) Interactions* |  |  |  |  |  |
| Cooks during the academic year x Family home | Doesn’t cook x city | Reference |  |  |  |
|  | Cooks x village | 0.47 | 0.320 | 0.37 | 0.441 |
| Tobacco use x Substance use | Doesn’t smoke x doesn’t use substances | Reference |  |  |  |
|  | Smokes x uses substances | 0.31 | 0.476 | 0.08 | 0.866 |
| m=number of subsets  BMI, body mass index; HPA, healthy physical activity; SES, socioeconomic status. | | | | | |

Table S6. Comparison between observed data and imputed data for unhealthy diet variable

|  | Unhealthy diet (%) | | | |
| --- | --- | --- | --- | --- |
| *Complete-case data* (n=593) |  | 47,89% |  |  |
| *Imputed data m=5* (n=924) | 1 | 48,64% |  |  |
|  | 2 | 51,36% |  |  |
|  | 3 | 50,45% |  |  |
|  | 4 | 49,55% |  |  |
|  | 5 | 43,20% |  |  |
| *Imputed data m=30* (n=924) | 1 | 42,60% | 16 | 47,43% |
|  | 2 | 47,13% | 17 | 49,55% |
|  | 3 | 52,87% | 18 | 48,64% |
|  | 4 | 45,32% | 19 | 50,76% |
|  | 5 | 51,36% | 20 | 46,83% |
|  | 6 | 49,24% | 21 | 45,62% |
|  | 7 | 50,45% | 22 | 49,85% |
|  | 8 | 47,13% | 23 | 51,66% |
|  | 9 | 49,85% | 24 | 48,64% |
|  | 10 | 48,34% | 25 | 48,94% |
|  | 11 | 47,73% | 26 | 44,11% |
|  | 12 | 51,36% | 27 | 46,22% |
|  | 13 | 48,04% | 28 | 50,15% |
|  | 14 | 53,47% | 29 | 46,22% |
|  | 15 | 54,38% | 30 | 45,02% |

m: number of subsets.

Table S7. Coefficients and standard error from multivariate logistic regression

|  |  | Complete-case analysis (n=593) | | Multiple imputation (n=924, m=5) | | Multiple imputation (n=924, m=30) | |
| --- | --- | --- | --- | --- | --- | --- | --- |
| Variables |  | Coefficient | SE | Coefficient | SE | Coefficient | SE |
| *a) Socioeconomic and demographic* |  |  |  |  |  |  |  |
| Age | 15-19 | Reference |  |  |  |  |  |
|  | 20-24 | -0,0005 | 0,1833 | -0,0763 | 0,1632 | 0,0201 | 0,1696 |
|  | 25-25+ | -0,0831 | 0,3537 | -0,0306 | 0,3263 | -0,0544 | 0,2992 |
| Gender | Woman | Reference |  |  |  |  |  |
|  | Man | 0,5580* | 0,1919 | 0,5858* | 0,1823 | 0,5749* | 0,1767 |
| SES father | Low | Reference |  |  |  |  |  |
|  | High | 0,2121 | 0,1839 | 0,1983 | 0,1641 | 0,1886 | 0,1739 |
| SES mother | Low | Reference |  |  |  |  |  |
|  | High | -0,4561* | 0,1889 | -0,4548* | 0,1725 | -0,4422* | 0,1813 |
| Family home | University city | Reference |  |  |  |  |  |
|  | Other town | 0,5232* | 0,1953 | 0,6344* | 0,1658 | 0,5261* | 0,1813 |
| Cooks for him or herself during the academic year | No | Reference |  |  |  |  |  |
|  | Yes | -0,0229 | 0,2022 | -0,1103 | 0,1926 | -0,0087 | 0,1959 |
| Degree course | Social Sciences | Reference |  |  |  |  |  |
|  | Health Sciences | -0,4616* | 0,2102 | -0,5193* | 0,195 | -0,4286* | 0,1827 |
| *b) Lifestyle-related* |  |  |  |  |  |  |  |
| BMI | Normal weight | Reference |  |  |  |  |  |
|  | Underweight | 0,8378* | 0,3678 | 0,7823* | 0,3628 | 0,824* | 0,3427 |
|  | Overweight | -0,2091 | 0,2322 | -0,3065 | 0,2751 | -0,2439 | 0,2256 |
|  | Obesity | 0,0403 | 0,4906 | 0,0388 | 0,4513 | 0,0263 | 0,4562 |
| HPA | No | Reference |  |  |  |  |  |
|  | Yes | -0,2000 | 0,1865 | -0,1995 | 0,1564 | -0,2179 | 0,1757 |
| Tobacco use | No | Reference |  |  |  |  |  |
|  | Yes | 0,1840 | 0,2335 | 0,1785 | 0,2169 | 0,194 | 0,2173 |
| Other harmful substances | No | Reference |  |  |  |  |  |
|  | Yes/occasional | 0,2533 | 0,2160 | 0,2839 | 0,1954 | 0,2492 | 0,2019 |
| * p<0.05. Complete-case analysis, multiple imputation where m= number of subsets.  Abbreviations: BMI, body mass index; HPA, healthy physical activity; SE: Standard error; SES: socioeconomic status | | | | | | |  |
